# Preparation and experimental evaluation of degradable esophageal stents based on novel polylactic acid materials

**DOI:** 10.3389/fbioe.2026.1827001

**Published:** 2026-06-11

**Authors:** Jianye Wang, Zhanhui Du, Xiao Zhu, Jiaxiang Tang, Qiteng Xu, Bei Lv, Bihui He, Quansheng Xing

**Affiliations:** 1 Qingdao University, Qingdao, Shandong, China; 2 Qingdao Women and Children’s Hospital, Qingdao University, Qingdao, Shandong, China; 3 Qingdao Hospital of Peking University People’s Hospital, Qingdao, Shandong, China

**Keywords:** compression molding, degradation property, esophageal stent, poly(lactide-co-caprolactone), radiopaque property

## Abstract

Esophageal stricture is a common esophageal disorder in both adults and children. The current standard clinical treatment is endoscopic dilation, which suffers from high recurrence rate and the need for repeated dilation. Although metallic esophageal stents can provide long-term support, they tend to cause complications such as migration, perforation, and bleeding, and require a second surgery for removal. Biodegradable stents are regarded as an ideal alternative because they can maintain esophageal patency and degrade spontaneously. To overcome the limitations of existing stents, this study used L-lactide and ε-caprolactone as main raw materials to prepare crosslinked polymers via ring-opening copolymerization and crosslinking reaction, and incorporated a contrast agent to construct a composite material with both imaging function and biodegradable properties. The material structure was characterized by Fourier-transform infrared spectroscopy, proton nuclear magnetic resonance spectroscopy, and gel permeation chromatography. Furthermore, an anti-migration stent structure was designed and fabricated into a solid stent by compression molding. *In vitro* mechanical performance tests and degradation experiments demonstrated that the prepared stent exhibits favorable mechanical properties and controllable degradability, showing potential application value in the treatment of benign and malignant esophageal strictures.

## 1 Introduction

The esophagus is a muscular conduit that connects the pharynx to the gastric cardia, serving as a critical organ for sustaining nutritional intake in the human body. A variety of benign and malignant conditions, including chronic inflammation and malignant tumors, can lead to stenosis or obstruction of the esophageal lumen (Okada et al., 2021; Yang et al., 2024). For malignant and refractory benign esophageal strictures, esophageal stent implantation has become the preferred treatment for relieving dysphagia and enhancing the patient’s quality of life (Spaander et al., 2021). However, traditional self-expandable metallic stents have obvious limitations in clinical application: long-term implantation is prone to complications such as migration, in-stent restenosis, and bleeding (Zhang Y. et al., 2025; Tabares Ocampo et al., 2023). Moreover, metallic stents are non-biodegradable and often require a second surgery for removal after implantation, which not only increases procedural risks but also adds to the burden on patients (Liu et al., 2022; Wang et al., 2023; Li et al., 2024).

With the development of tissue engineering and molecular materials science, biodegradable stents have emerged. Such stents exhibit excellent histocompatibility and can degrade completely after being implanted *in vivo* for a period of time, thus avoiding many complications associated with traditional metallic stents (Zhang Y. et al., 2025). Biodegradable esophageal stents have also become one of the current research hotspots in the field of esophageal stents. At present, there is a wide variety of biodegradable materials, among which polylactic acid (PLA), polycaprolactone (PCL) and others have been widely used in the medical field. Polylactic acid and polycaprolactone possess good biocompatibility, and their final degradation products in the human body are water and carbon dioxide, which are safe and non-toxic (Li et al., 2023), have been certified as safe materials by the United States Food and Drug Administration (FDA) (Reshma et al., 2024; Pan et al., 2025; Zhang S. et al., 2025). However, polylactic acid (PLA) has drawbacks such as poor ductility and slow degradation, which limit its application as a single material (Pan et al., 2025); However, it exhibits excellent mechanical properties, and its toughness and hydrophobicity can be further improved through copolymerization or blending modification (Tiwari et al., 2023; Yan et al., 2024; Li et al., 2025). In contrast, polycaprolactone (PCL) has good ductility and a high elongation at break, and the materials prepared from it exhibit better flexibility and impact resistance (Ye et al., 2024). Studies have demonstrated that the introduction of polycaprolactone (PCL) into the polylactic acid (PLA) system can effectively improve its properties. At present, their copolymers have been widely used in drug delivery systems, tissue engineering scaffolds, cardiovascular materials and other fields (Kim et al., 2025; Huang et al., 2025; Fujimoto et al., 2021). However, there are few studies on the application of their copolymers in esophageal stents. Moreover, most esophageal stents prepared from existing biodegradable materials exhibit poor imaging performance. Based on the above, this study develops an esophageal stent with both imaging capability and biodegradable properties, which can provide a novel strategy for the clinical treatment of esophageal stenosis. In this study, iodine-containing diol, namely, diiodoneopentyl glycol (DINPG), was selected as a contrast agent. Its dihydroxyl structure in the molecule can participate in cross-linking reactions to achieve chemical bonding. Meanwhile, iodine atoms provide radiopacity, and the neopentyl glycol skeleton endows it with nucleation and toughening effects, thus realizing dual functions of radiopacity enhancement and mechanical reinforcement.

## 2 Materials and methods

### 2.1 Experimental materials

L-lactide, ε-caprolactone, isophorone diisocyanate, sodium iodide, and dibromoneopentyl glycol were procured from Adamas Reagent Co., Ltd., Shanghai. Zinc neodecanoate was obtained from Sigma-Aldrich. Pentaerythritol was sourced from Laibosite Technology Co., Ltd. (Qingdao, China). L929 cells (murine fibroblasts) were acquired from iCell Biotechnology Co., Ltd., Shanghai. Phosphate-buffered saline (PBS) was obtained from ProCell Life Science and Technology Co., Ltd. (Wuhan, China). Trypsin was sourced from Biosharp Life Sciences (Beijing, China). The CCK-8 reagent was purchased from Invitrogen.

### 2.2 Preparation and characterization of poly(lactide-co-caprolactone) (PLCL)

#### 2.2.1 Preparation of PLCL

**SCHEME 1:**
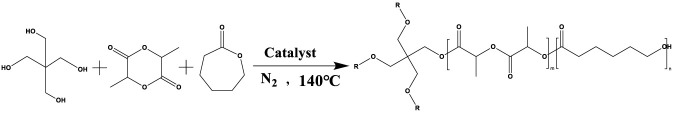
Synthetic route of PLCL.

Firstly, a two-neck reaction flask was prepared and a magnetic stir bar was placed inside. The flask was then sealed with a glass stopper and a rubber septum, and vacuumized using a double-tube manifold system. At room temperature, 15 g of L-lactide and 35 g of ε-caprolactone were weighed for later use. The magnetic heating stirrer was turned on, and the oil bath temperature was set to 140 °C. The two prepared monomers were added into the reaction flask, followed by the catalyst zinc neodecanoate and initiator pentaerythritol. The flask was purged with nitrogen to eliminate air interference, and then immersed in silicone oil for a 48-h oil bath reaction. Upon completion of the reaction, the mixture was cooled to room temperature, and the product was collected to obtain the crude PLCL. The crude product was then purified using tetrahydrofuran and n-hexane to obtain highly pure PLCL, and the synthetic route is shown in Scheme 1.

#### 2.2.2 Fourier transform infrared Spectroscopy (FTIR)

The chemical structure of PLCL in the wavenumber range of 400–4,000 cm^-1^ was analyzed using a Nicolet 6700 FTIR spectrometer produced by Thermo Fisher Scientific (United States). The spectrometer had a resolution of 4 cm^-1^, a signal-to-noise ratio of 50,000:1, and was operated with 32 scans.

#### 2.2.3 Proton nuclear magnetic resonance spectroscopy

The composition and structure of PLCL were analyzed using a Bruker AVANCE III 600 MHz nuclear magnetic resonance (NMR) spectrometer (Bruker Corporation, Germany). The samples were dissolved in dimethyl sulfoxide (DMSO) with tetramethylsilane as the internal standard.

#### 2.2.4 Gel permeation chromatography

The molecular weights and molecular weight distributions of PLCL were analyzed using an APL-GPC220 gel permeation chromatography (GPC) system from Agilent Technologies, United States THF served as the mobile phase, with a test temperature of 40 °C, and polystyrene was utilized as the reference standard.

### 2.3 Preparation and characterization of cross-linked PLCL

#### 2.3.1 Preparation of crosslinked PLCL

**SCHEME 2:**
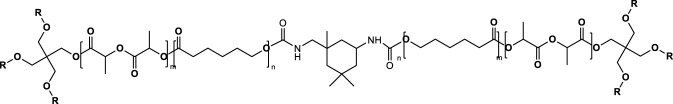
Chemical structure of cross-linked PLCL.

4 g of purified PLCL and 0.44 g of isophorone diisocyanate were weighed out. After adding zinc neodecanoate as the catalyst, the mixture was stirred thoroughly to achieve homogeneity. The system was then placed in an oven at 45 °C for 16 h to yield cross-linked PLCL, and its chemical structure is shown in Scheme 2.

#### 2.3.2 FTIR

The chemical structure of cross-linked PLCL in the wavenumber range of 400–4,000 cm^−1^ was analyzed using a Nicolet 6700 FTIR spectrometer produced by Thermo Fisher Scientific (United States). The spectrometer featured a resolution of 4 cm^−1^, a signal-to-noise ratio of 50000:1, and was operated with 32 scans.

### 2.4 Preparation and characterization of diiodoneopentyl glycol

#### 2.4.1 Preparation of diiodoneopentyl glycol

**SCHEME 3:**
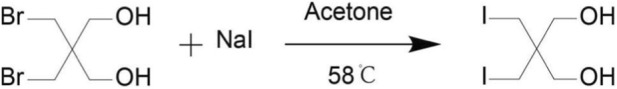
Synthetic route of diiodoneopentyl glycol (DINPG).

Firstly, 15 g of dibromoneopentyl glycol and 20 g of sodium iodide were weighed into a reaction flask, and 100 mL of anhydrous acetone was added. The mixture was stirred until the raw materials were completely dissolved. Subsequently, the reaction system was heated to 58 °C and stirred under reflux for 4 days. After completion of the reaction, the supernatant in the flask was transferred to a new reaction flask. Next, 8 g of sodium iodide and 20 mL of anhydrous acetone were added to the new flask, and the reaction was continued at 58 °C with stirring for another 4 days. After the reaction, acetone in the supernatant was removed by rotary evaporation to obtain a pale yellow crude product. The crude product was then extracted with ethyl acetate and sodium thiosulfate solution to afford white crystals of diiodoneopentyl glycol, and the synthetic route is shown in Scheme 3.

#### 2.4.2 FTIR

The chemical structure of diiodoneopentyl glycol in the wavenumber range of 400–4,000 cm^−1^ was analyzed using a Nicolet 6700 FTIR spectrometer produced by Thermo Fisher Scientific (United States). The spectrometer featured a resolution of 4 cm^−1^, a signal-to-noise ratio of 50000:1, and was operated with 32 scans.

### 2.5 Preparation and mechanical properties characterization of novel polylactic acid-based materials and esophageal stents

#### 2.5.1 Preparation of the novel polylactic-acid-based material

4 g of purified PLCL and 44 mg of isophorone diisocyanate were weighed, followed by the addition of 0.8 g of diiodoneopentyl glycol and zinc neodecanoate as the catalyst. The mixture was stirred thoroughly until homogeneous, and then placed in an oven at 45 °C for 16 h to obtain the novel polylactic acid-based material.

#### 2.5.2 Mold preparation and fabrication of esophageal stents

The esophageal stent was designed using UG NX 2306 software, and the design drawings were then sent to a mold manufacturing factory to produce the physical mold. 4 g of purified poly(lactide-co-caprolactone) and 44 mg of isophorone diisocyanate were weighed, followed by the addition of 0.8 g of diiodoneopentyl glycol and zinc neodecanoate as the catalyst. The mixture was stirred thoroughly until homogeneous, then poured into the esophageal stent mold. The mold was placed in an oven at 45 °C for 16 h to complete the reaction. After the reaction, the mold was taken out and cooled to room temperature. The mold was opened, and the esophageal stent was removed to obtain the final product.

#### 2.5.3 Tensile test

Tensile testing was performed on type 1A specimens prepared according to ISO 527-2:2012 using an LD24.204 universal testing machine from Shanghai Lishi, at a crosshead speed of 10 mm/min.

#### 2.5.4 Radial support force test

The radial support force of the esophageal stent was measured using an LD24.204 universal testing machine from Shanghai Lishi. The sample was placed parallel between two plates and gradually compressed. When the distance between the plates was reduced to 1/2 of the original diameter of the sample, the pressure value recorded by the universal testing machine was defined as the radial support force of the sample.

#### 2.5.5 Anti-migration force test

The anti-migration force of the stent was tested using a simplified esophageal model made of silica gel with an LD24.204 universal testing machine from Shanghai Lishi. The lower end of the simplified esophageal model was fixed by the testing machine, and the esophageal stent was inserted into the model. A hole was drilled at the upper end of the stent and tied to the testing machine with a string. The esophageal stent was pulled out uniformly from the model by the testing machine. The force-displacement curve and the peak force during the uniform axial pulling of the stent were recorded.

#### 2.5.6 Compression recovery test

The esophageal stent samples were tested using a self-made esophageal delivery system. The system consisted of a silica gel hose with an inner diameter of 12 mm and an outer diameter of 14 mm, as well as a delivery tip. The initial inner diameters at both ends and the waist of the stent were measured first. The stent was then placed into the delivery system and held for 3 min, after which it was pushed out and the recovered inner diameters at both ends and the waist were measured immediately. Five stents were used for repeated tests. The inner diameter recovery rate of the stent was calculated as follows: Recovery rate (%) = (Recovered inner diameter/Initial inner diameter) × 100%.

### 2.6 Other property characterization of novel polylactic acid-based materials and esophageal stents

#### 2.6.1 Static water contact angle measurement

The static water contact angle of the test samples was measured using an optical contact angle and 3D topography measurement system from Biolin, Sweden.

#### 2.6.2 Scanning electron microscope

The surface and fracture morphologies of the test samples were observed using an Apreo C field-emission scanning electron microscope from Thermo Fisher Scientific, United States. The material films were immersed in liquid nitrogen for brittle fracture. The surfaces and fracture surfaces were sputter-coated with gold using a Hitachi MC1000 ion sputter coater prior to observation. The imaging voltage was 5 kV.

#### 2.6.3 Development performance test

The test samples and high-purity aluminum sheets were scanned and imaged using a small-animal Micro CT from Jiangsu Pusheng Technology. The test samples were material sheets with dimensions of 1.0 cm × 1.0 cm × 0.2 cm, and the high-purity aluminum sheets had the same specifications as the material sheets. The scanning voltage was 90 kV. The test specimens and high-purity aluminum sheets were imaged using the Runyes X-ray machine RAY98(p).

#### 2.6.4 *In vitro* cytotoxicity test

Cell Culture: L929 cells were used as experimental cells, and the cytotoxicity of the material was determined by the CCK-8 method. L929 cells were cultured in high-glucose DMEM medium (89% medium + 10% fetal bovine serum +1% penicillin/streptomycin) in a constant-temperature incubator at 37 °C with 5% CO_2_. When the cells reached 80%–90% confluence, they were digested with 0.25% trypsin, collected by centrifugation, resuspended in high-glucose DMEM medium, and subcultured into two new cell culture flasks. The cells were cultured in the incubator until the growth density reached 60%–70%.

Preparation of Material Extracts: Test samples and polyethylene films were prepared into specimens of 1.0 cm × 1.0 cm × 0.2 cm according to ISO 10993 and sterilized by ethylene oxide. The materials were immersed in high-glucose DMEM medium (89% medium +10% fetal bovine serum +1% penicillin/streptomycin) at a ratio of sample surface area to extraction medium of 3 cm^2^/mL. After incubation at 37 °C for 72 h, extracts of the novel polylactic acid-based material and polyethylene material were obtained.

CCK-8 Assay: L929 cells in the logarithmic growth phase were collected and seeded into 96-well plates at a density of 4 × 10^4^ cells per well. The extract of the novel polylactic acid-based material was diluted to 25%, 50%, and 100% of the original concentration as the experimental groups, while the polyethylene extract served as the negative control group. Cells in all groups were incubated at 37 °C with 5% CO_2_ for 72 h. Subsequently, 100 μL of CCK-8 solution was added to each well, and incubation was continued for another 2 h. The absorbance of each sample was measured at a wavelength of 450 nm using a SPARK 10 M microplate reader (Tecan, Switzerland). The cytotoxicity of the material was calculated as follows: Cell viability (%) = (Absorbance of experimental group/Absorbance of negative control group) × 100%.

#### 2.6.5 Degradation test

Three esophageal stents with identical specifications were chosen, and their weights and radial compressive strengths were assessed at room temperature. The stents were then placed in PBS at a consistent temperature of 37 °C for an 8-week observation period. Every 2 weeks were taken out, washed thrice with deionized water, and dried until reaching a constant weight at room temperature. Subsequently, their weights and radial compressive strengths were measured again. The rate of mass loss for the samples was determined using the formula: mass-loss rate (%) = (weight loss of the stent/initial weight of the stent) × 100.

## 3 Results and discussion

### 3.1 Preparation results of cross-linked PLCL

PLCL was synthesized via ring-opening copolymerization (mass ratio 3:7) with low-toxicity zinc neodecanoate. Crosslinked PLCL was prepared using IPDI as crosslinker.

#### 3.1.1 Detection results of PLCL

**FIGURE 1:**
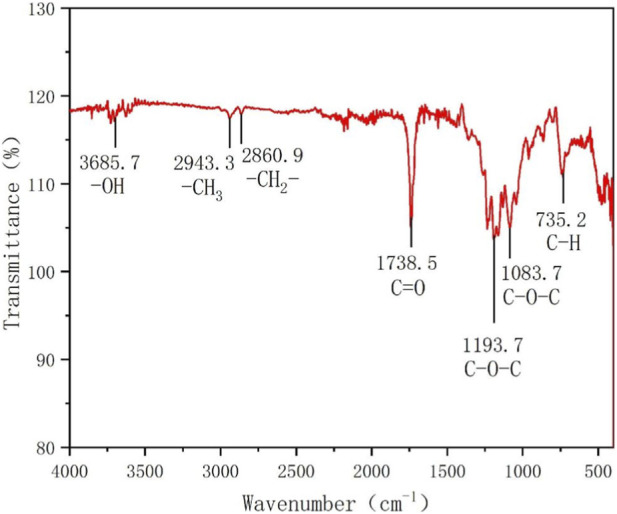
FTIR of PLCL.

First, the chemical structure of PLCL was analyzed by FTIR, and the results are shown in Figure 1. The infrared absorption spectrum shows an -OH absorption peak at 3,685.7 cm^−1^, indicating the presence of hydroxyl groups at the ends of the molecular chains. The absorption peaks at 2,943.3 cm^−1^ and 2,860.9 cm^−1^ are attributed to -CH_3_ and -CH_2_-, respectively. A stretching vibration peak of C=O appears at 1738.5 cm^−1^, and asymmetric and symmetric stretching vibration peaks of C-O-C are observed at 1,193.7 cm^−1^ and 1,083.7 cm^−1^, which represent the formation of ester linkages. The existence of the above characteristic absorption peaks confirms that L-lactide, ε-caprolactone, and pentaerythritol underwent copolymerization, and the target product poly(lactide-co-caprolactone) was successfully synthesized.

**FIGURE 2:**
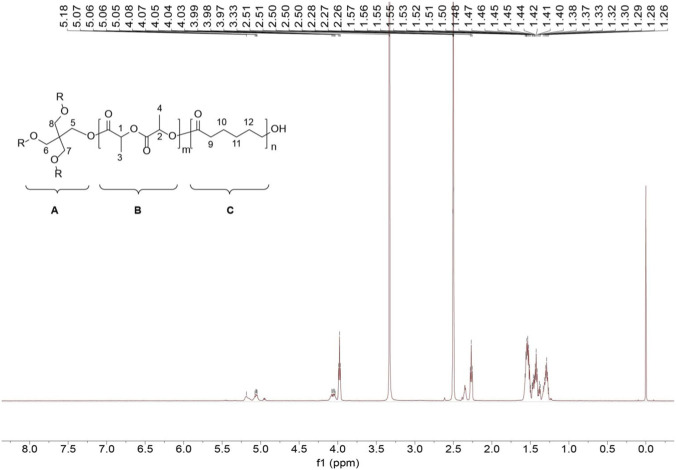
^1^H NMR spectrum of PLCL.

The molecular structure of poly(lactide-co-caprolactone) was further characterized by proton nuclear magnetic resonance spectroscopy, and the results are shown in Figure 2. ^1^H-NMR (600 MHz, DMSO-d_6_) δ 5.18 (s, H-1), 5.06 (dd, J = 10.7, 6.8 Hz, H-2), 4.05 (dt, J = 11.7, 6.1 Hz, H-5), 3.98 (t, J = 6.6 Hz, H-6,7,8), 2.27 (t, J = 7.3 Hz, H-9), 1.54 (tq, J = 15.0, 7.2 Hz, H-10,11), 1.48–1.37 (m, H-12), 1.29 (p, J = 7.8 Hz, H-3,4). Through spectral data analysis and peak assignment of PLCL, the distinctive peaks of segment A were identified at 4.05 (dt, J = 11.7, 6.1 Hz, H-5) and 3.98 (t, J = 6.6 Hz, H-6,7,8), respectively; the characteristic peaks of segment B were detected at 5.18 (s, H-1), 5.06 (dd, J = 10.7, 6.8 Hz, H-2) and methyl protons at 1.29 (p, J = 7.8 Hz, H-3,4); the distinctive peaks of segment C were observed at 2.27 (t, J = 7.3 Hz, H-9), 1.54 (tq, J = 15.0, 7.2 Hz, H-10,11), and 1.48–1.37 (m, H-12). The appearance of the above characteristic signals further confirms the successful synthesis of PLCL.

The relative molecular weight and distribution of the samples were further analyzed by gel permeation chromatography (GPC), and the results are shown in Table 1. The theoretical number-average molecular weight (Mn) of PLCL was 40,000, while the experimentally determined (Mn) was 40,487, closely aligning with the theoretical value. In this study, the ratio of Mw (55,190) to Mn (40,487), namely, the polydispersity index (PDI = 1.363), falls within a narrow range (PDI values of conventionally synthesized polymers are generally 1.5–2.0). This indicates that the synthesized poly(lactide-co-caprolactone) possesses a narrow molecular weight distribution and uniform chain-length homogeneity. Such a narrow distribution helps ensure stable material performance across different batches, preventing premature degradation caused by excessive low-molecular-weight fractions or processing difficulties induced by high-molecular-weight fractions. The higher Mw relative to Mn is a normal outcome of mass-weighted averaging. Together, these two parameters verify that the polymer achieves the target molecular weight (theoretical Mn = 40,000) and exhibits molecular weight properties suitable for subsequent cross-linking modification and compression molding.

**TABLE 1:** Molecular weight and molecular weight distribution of PLCL.

Mn	Mw	PDI
40,487	55,190	1.363

#### 3.1.2 Detection results of cross-linked PLCL

**FIGURE 3:**
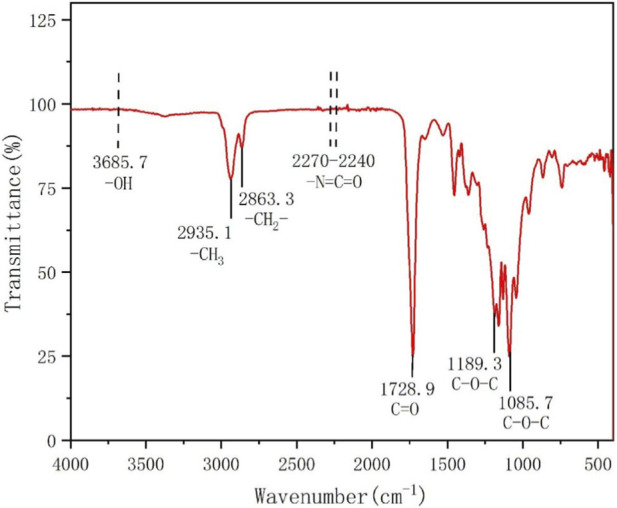
FTIR of crosslinked PLCL.

The chemical structure of cross-linked poly(lactide-co-caprolactone) was characterized by FTIR, and the results are shown in Figure 3. The infrared absorption peak curve displays peaks at 2,935.1 cm^−1^ and 2,863.3 cm^−1^, corresponding to –CH_3_ and –CH_2_ groups, respectively. A stretching vibration peak of C=O is evident at 1728.9 cm^−1^, while asymmetric and symmetric stretching vibration peaks of C–O–C are identified at 1,189.3 cm^−1^ and 1,085.7 cm^−1^, respectively, indicating the presence of ester groups in the copolymer. Notably, no absorption peak of –N=C=O (the characteristic peak of isophorone diisocyanate) is detected in the range of 2,270–2,240 cm^−1^, and the–OH absorption peak at 3,685.7 cm^−1^ is absent. These phenomena indicate that the isocyanate groups of isophorone diisocyanate reacted completely with the hydroxyl groups at the chain ends of poly(lactide-co-caprolactone), and a urethane cross-linked structure was successfully formed. Based on the changes in the above characteristic absorption peaks, the successful synthesis of cross-linked poly(lactide-co-caprolactone) can be confirmed.

### 3.2 Preparation of the developer diiodoneopentyl glycol

**FIGURE 4:**
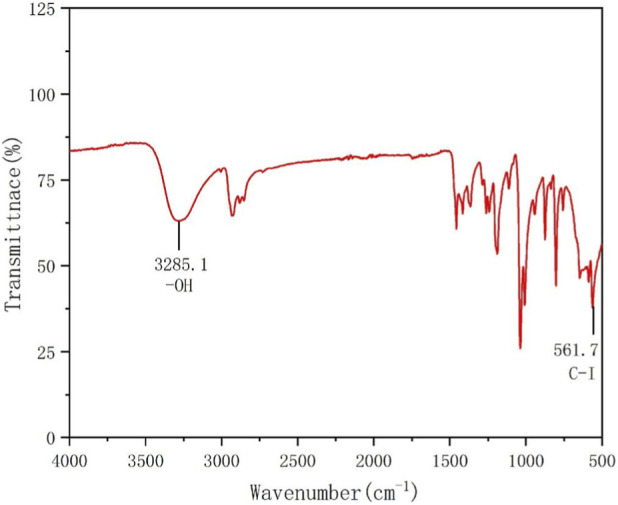
FTIR of DINPG.

To endow the scaffold fabricated from cross-linked PLCL with radiopacity, an iodine-containing functional monomer, diiodoneopentyl glycol, was synthesized via nucleophilic substitution reaction between dibromoneopentyl glycol and sodium iodide, and used as a contrast agent for the polymer. The FTIR result of diiodoneopentyl glycol is shown in Figure 4. A strong and broad -OH absorption peak appears at 3,285.1 cm^−1^, indicating that the product possesses the basic structure of neopentyl glycol. In the fingerprint region of 650–500 cm^−1^, the strongest peak is centered at 561.7 cm^−1^, which is below 600 cm^−1^ and shifts toward lower wavenumbers. This peak is assigned to the C-I absorption, confirming the successful synthesis of diiodoneopentyl glycol.

### 3.3 Preparation results of esophageal stents

**FIGURE 5:**
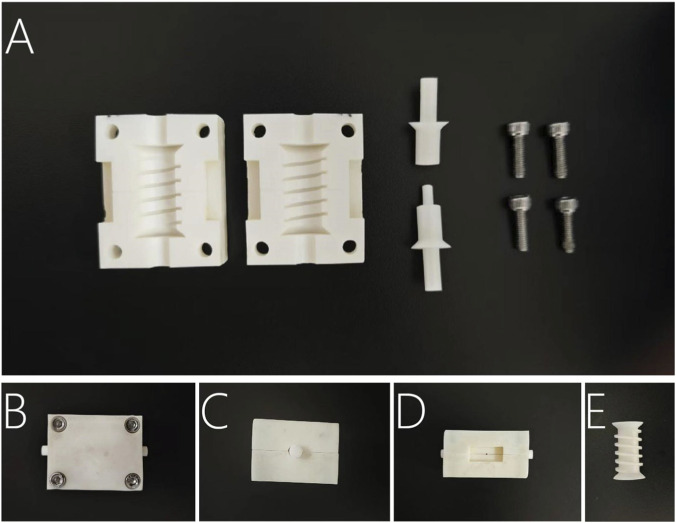
Mold assembly and final esophageal stent: **(A)** mold parts; **(B)** top view; **(C)** side view; **(D)** front view; **(E)** demolded stent.

For the material preparation of the stents, the contrast agent diiodoneopentyl glycol was introduced during the preparation of cross-linked poly(lactide-co-caprolactone), and a composite material with radiopacity, namely, the novel polylactic acid-based material, was finally obtained. For the structural design of the stents, a bell-shaped opening at both ends and an external spiral texture at the waist were designed based on the conventional straight tubular esophageal stent. Literature studies have shown that both structures can effectively enhance the anchoring force between the stent and the esophageal wall, and significantly reduce the risk of stent migration (Garbey et al., 2016; Lin et al., 2019). The stent was fabricated using a compression molding process. The aforementioned novel polylactic acid-based material was processed into a solid esophageal stent via a mold. This process offers the advantage of fabricating stents with various complex structures, enabling personalized customization and mass production of esophageal stents. The photographs of the mold and the stent are shown in Figure 5.

**FIGURE 6:**
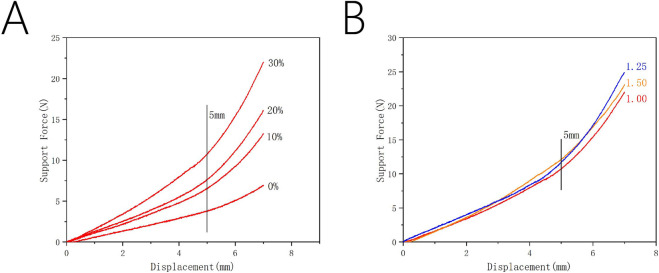
Radial support force: **(A)** effect of DINPG content; **(B)** effect of crosslinker dosage.

**SCHEME 4:**
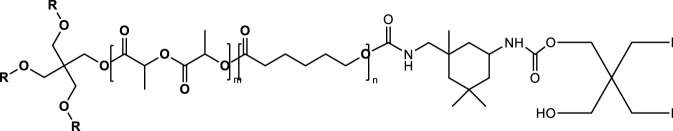
DINPG incorporation into crosslinked PLCL network.

**FIGURE 7:**
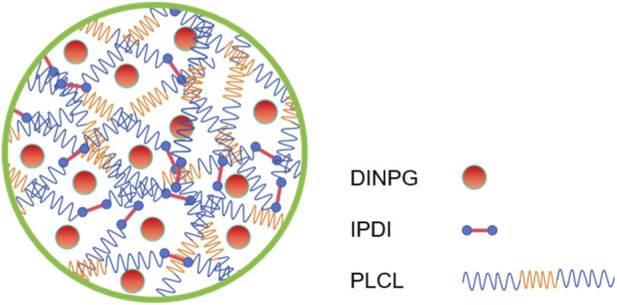
Schematic of DINPG as crosslinking comonomer and nucleating agent.

Since both the mechanical properties and radiopacity of the stent are affected by the content of the contrast agent, four types of esophageal stents were systematically prepared in this study. The mass fractions of the contrast agent were 0%, 10%, 20%, and 30% relative to the PLCL matrix, respectively. The optimal contrast agent content was determined by comparing the radial supporting forces of the stents with different ratios, and the results are shown in Figure 6A. Within the contrast agent mass fraction range of 0%–30%, the radial supporting force of the stent showed a significant increasing trend with the increase of the contrast agent content. The mechanism of this phenomenon can be explained from the following two aspects: First, the contrast agent diiodoneopentyl glycol contains hydroxyl groups, which can participate in the cross-linking reaction and be embedded into the network structure formed by cross-linked PLCL, making the network structure more stable, and tougher, and the reaction equation is presented in Scheme 4. Second, as shown in Figure 7, as a small-molecule substance, diiodoneopentyl glycol can act as a nucleating agent and fill the voids in the network structure, resulting in a denser overall structure of the stent. Such changes in the microstructure are macroscopically reflected in the significantly enhanced radial supporting force of the stent. In summary, the introduction of the contrast agent diiodoneopentyl glycol not only endows the stent with the required radiopacity, but also further improves its radial supporting force and structural stability, providing a more reliable mechanical guarantee for its clinical application.

It should be noted that the void capacity of the network structure formed by cross-linked PLCL is limited. It was found experimentally that when the content of the contrast agent exceeds 30% of the mass of PLCL, the contrast agent cannot be fully incorporated into the composite system. This phenomenon indicates that a loading of 30% is close to or reaches the maximum capacity limit of the cross-linked network for DINPG, meaning that the voids in the network structure are basically filled. On this basis, 30% was determined as the optimal loading ratio of the contrast agent in this study, at which the stent exhibits both satisfactory radiopacity and structural integrity.

In addition, hydroxyl groups from moisture in ambient air may react with isophorone diisocyanate, the cross-linking agent, resulting in a relative decrease in its effective concentration and further interfering with the completion of the target cross-linking reaction. To compensate for this loss, the actual addition ratio of the cross-linking agent was appropriately increased relative to its theoretical dosage. The optimal addition ratio was investigated by comparing the radial supporting forces of stents prepared with 1.00, 1.25, and 1.50 times the theoretical dosage of the cross-linking agent. The experimental results are shown in Figure 6B. As the dosage of the cross-linking agent increased, the radial supporting force of the stent showed a clear increasing trend, confirming that moisture in the air exerted a negative effect on the cross-linking reaction, and a moderate increase in cross-linker dosage could offset this effect. Further analysis revealed that the radial supporting forces of stents prepared with 1.25 and 1.50 times the theoretical cross-linker dosage were similar. Given the high toxicity of isophorone diisocyanate, excessive addition would significantly compromise the biosafety of the stent. Therefore, considering both mechanical performance and biosafety, 1.25 times the theoretical dosage was determined as the optimal addition ratio of the cross-linking agent. Solid stents were fabricated according to the above-determined ratios of contrast agent (30 wt%) and cross-linker (1.25 times the theoretical amount), and their radial supporting force was approximately 11.7 N. According to literature reports, the radial force of commercially available esophageal stents ranges from 3.6 N to 11.5 N with a diameter of approximately 10 mm, while the radial force of biodegradable esophageal stents is generally below 10 N. Therefore, the supporting performance of the esophageal stents prepared in this study exceeds that of biodegradable esophageal stents and commercial metallic stents with similar specifications (Hanbo, 2018; Tanaka et al., 2006; Hirdes et al., 2013), meeting the clinical requirements for stent supporting performance.

**FIGURE 8:**
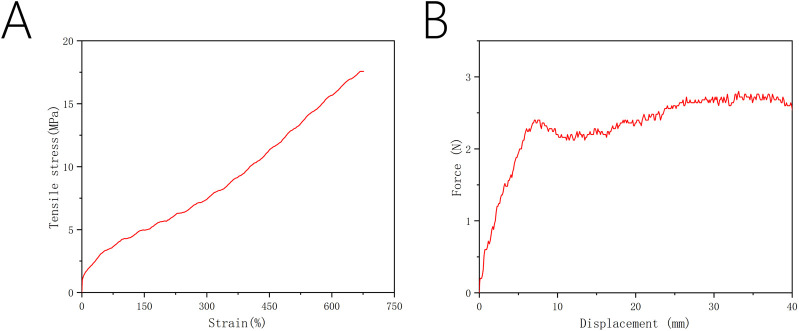
Mechanical performance: **(A)** tensile curve of composite; **(B)** anti-migration force curve.

**FIGURE 9:**
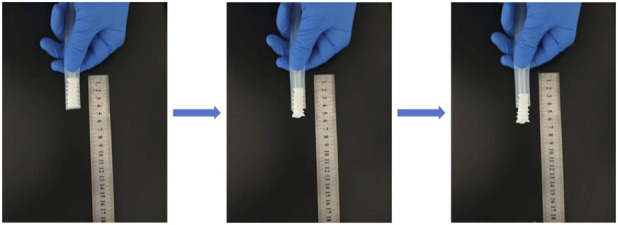
*In vitro* compression–release simulation of the esophageal stent.

On the premise of sufficient radial support force, esophageal stents also need excellent elastic recovery performance to enable smooth loading into the delivery system and rapid restoration to their original shape after deployment at the stenotic site *in vivo*. This is a critical prerequisite for the clinical implantation and functional performance of stents. Accordingly, in this study, the mechanical and elastic properties of the novel polylactic acid-based material were first characterized via tensile tests, with the results presented in Figure 8A. The tensile strength of the material was approximately 17.57 MPa, and the elongation at break was about 638.43%. Compared with metallic materials, the novel polylactic acid-based material exhibits relatively lower tensile strength but significantly superior elongation at break. This indicates that it exhibits more excellent elasticity, flexibility and shape recovery ability. Stents fabricated from this material can more easily return to their original shape upon deployment at the stenotic site (Sun et al., 2025; Long et al., 2023). We further investigated the actual shape recovery capability of the stents via compression-release tests, and the release behavior is shown in Figure 9. The experimental results are listed in Table 2. For the five stents tested, the inner diameter recovery ratios at both the waist and the two ends all exceeded 98%. These results fully confirm that the as-prepared novel polylactic acid-based esophageal stents can withstand the compressive loading similar to that encountered during clinical implantation, and can steadily recover to their original shape after compression. This indicates that the stents possess favorable mechanical stability without permanent deformation or structural damage throughout the implantation procedure, thus meeting the mechanical requirements for clinical applications.

**TABLE 2:** Results of the compression recovery test.

Stent number	Initial waist inner diameter (mm)	Recovered waist inner diameter (mm)	Waist inner diameter recovery rate (%)	Initial inner diameter of both ends (mm)	Recovered inner diameter of both ends (mm)	Inner diameter recovery rate of both ends (%)
1	10	9.88	98.8	15	14.80	98.7
2	10	9.95	99.5	15	14.92	99.5
3	10	9.84	98.4	15	14.81	98.7
4	10	9.96	99.6	15	14.92	99.5
5	10	9.83	98.3	15	14.87	99.1

Esophageal stents should provide effective radial support at the esophageal stenotic site, and meanwhile possess certain anti-migration properties to reduce the risk of stent displacement after implantation. Such performance can usually be achieved by modifying the stent structure. Previous studies have demonstrated that the migration resistance of stents can be predicted by the anti-migration force (Brinkmann et al., 2024). The higher the anti-migration force, the greater the migration resistance of the stent, and the lower the probability of displacement, thereby reducing various complications associated with stent migration. On this basis, the anti-migration performance of the designed stent structure was evaluated in this study through anti-migration force testing. The results are shown in Figure 8B. The force-displacement curve exhibited an upward trend at the initial stage and then gradually became relatively stable. The test results showed that the maximum anti-migration force of the esophageal stent was approximately 2.8 N, indicating that the stent possessed favorable anti-migration performance and could reduce the risk of stent displacement. In addition, compared with metallic stents, biodegradable stents can degrade spontaneously after providing temporary support, which fundamentally eliminates the risks of long-term implantation-induced delayed migration, tissue ingrowth, and restenosis (Zhang Y. et al., 2025), further improving the safety of their clinical applications. In this study, pull-out tests were conducted using a simplified esophageal model fabricated from silicone. Featuring a straight tubular structure, this model cannot fully replicate the physiological curvature, peristaltic contraction of the human esophagus, or the lubricating effect of mucosal secretions. Studies have demonstrated that static pull-out tests mainly reflect the pure frictional anchoring force between the stent and the esophageal wall, while failing to adequately evaluate the anti-migration performance of stents during dynamic swallowing. In a dynamic physiological environment, the axial compliance and bending stiffness of stents may exert a more significant impact on migration resistance. Despite these limitations, static pull-out force testing remains one of the standard *in vitro* methods internationally used to assess the anti-migration performance of esophageal stents. With its simple operation and good repeatability, this testing method is suitable for material screening and structural optimization in the early design stage of stents. Using this test, the present study verified the anti-migration effects of the flared end and spiral groove design, providing preliminary evidence for subsequent animal experiments.

### 3.4 Other performance test results of the material and the stent

#### 3.4.1 Static water contact angle measurement

**FIGURE 10:**
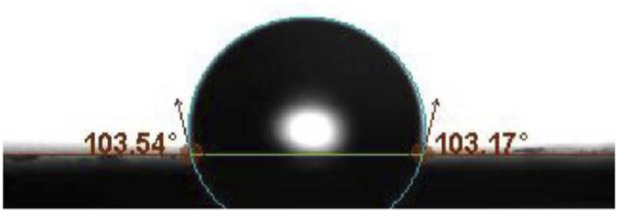
Static water contact angle measurement.

The hydrophilicity and hydrophobicity of a material can be determined by water contact angle measurement. Generally, a hydrophilic surface is defined as having a water contact angle <90°, while a hydrophobic surface has a water contact angle >90°. In this study, the surface wettability of the novel polylactic acid-based material was evaluated, and the results are shown in Figure 10. The static water contact angle of the material surface was 103.355° ± 0.185°, which was significantly larger than 90°, indicating an obvious hydrophobic characteristic. Within the esophageal environment, esophageal stents must contend with fibroblast activation, inflammatory responses, and scar formation induced by mechanical irritation. According to literature, hydrophobic surfaces significantly enhance the adsorption of serum proteins via hydrophobic interactions, forming a protein adsorption layer that acts as a bioinert barrier, which is unfavorable for cell adhesion (Arima and Iwata, 2007; Arima and Iwata, 2015). From the perspective of tissue healing, hydrophobic surfaces generally exhibit poor cell adhesion and proliferation properties. Theoretically, this may inhibit excessive infiltration of fibroblasts, thereby delaying scar formation and reducing the incidence of recurrent esophageal stenosis (Yang et al., 2024; Ferrari et al., 2019; Jun et al., 2018; Ozdol et al., 2007). Therefore, the esophageal stent fabricated from the novel polylactic acid-based material can not only provide stable support for the stenotic esophageal segment, but also further reduce the risk of esophageal restenosis through its inherent hydrophobic property, which enhances the clinical application value of the stent.

#### 3.4.2 Radiopacity test

**FIGURE 11:**
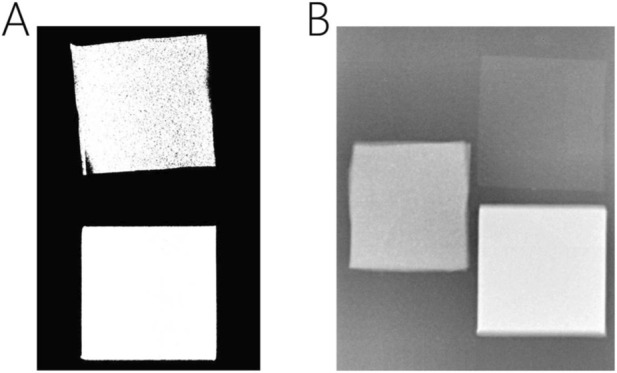
Radiopacity Test. **(A)** CT scan; **(B)** X-ray fluoroscopy.

To verify the radiopacity of the novel polylactic acid-based material, computed tomography (CT) and X-ray fluoroscopy were employed in this study to evaluate its imaging performance. As shown in Figure 11A, the CT images of the test specimen (upper) and the high-purity aluminum sheet (lower) were acquired at a voltage of 90 kV. The results revealed that the average CT value of the high-purity aluminum sheet was approximately 3167 HU, while that of the test specimen was about 1579 HU. It is known that the CT values of cortical bone in healthy adults at 90 kV mostly range from 1,500 to 2500 HU. The CT value of the test specimen falls within this range, and its CT radiopacity is close to that of cortical bone in healthy adults. On the basis of CT scanning, X-ray fluoroscopy was further conducted to assess the radiopacity of the material. As illustrated in Figure 11B, the test specimen fabricated from the novel polylactic acid-based material is shown on the left, the specimen made of cross-linked poly(lactide-co-caprolactone) is shown in the upper right, and the high-purity aluminum sheet is shown in the lower right, with all three samples having identical dimensions. It can be observed that the radiopacity of the novel polylactic acid-based material is weaker than that of the high-purity aluminum sheet, yet significantly superior to that of cross-linked poly(lactide-co-caprolactone). This result is consistent with the CT findings, confirming that the incorporation of the contrast agent diiodoneopentyl glycol endows the material with favorable radiopacity. The above results indicate that the novel polylactic acid-based material exhibits excellent radiopacity. Esophageal stents prepared from this material can achieve radiopacity comparable to that of adult cortical bone, meeting the clinical requirements for stent visualization and facilitating postoperative monitoring of stent position and status.

#### 3.4.3 Scanning electron microscope

**FIGURE 12:**
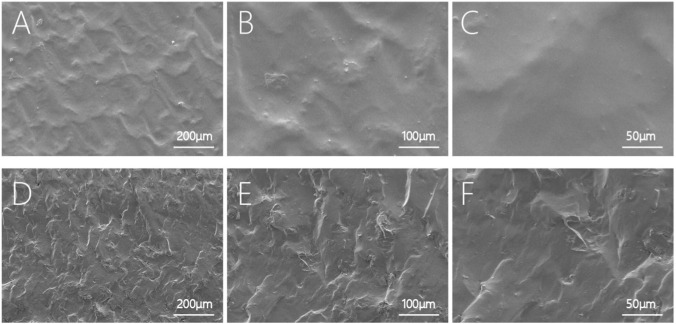
SEM micrographs: **(A–C)** surface at 200, 100, 50 μm; **(D–F)** fracture surface at 200, 100, 50 μm.

The surface and fracture morphologies of the novel polylactic acid-based material were observed by scanning electron microscopy to evaluate its microstructural uniformity, interfacial compatibility, and structural integrity, which are closely correlated with mechanical stability, radial support performance, and long-term service safety. As shown in Figure 12, at low magnification (200 μm), the material surface was smooth and uniform with low roughness, and no obvious defects such as cracks, pores, or phase separation were observed. At medium and high magnifications (100 μm and 50 μm), the surface remained compact and homogeneous, indicating good compatibility between the cross-linked PLCL matrix and diiodoneopentyl glycol. The fracture surface exhibited a ductile fracture morphology with moderate roughness, without microcracks, voids, or structural collapse. The uniformly distributed contrast agent domains were tightly embedded in the polymer network, forming a dense and integrated microstructure. These features confirm that: The contrast agent diiodoneopentyl glycol was well dispersed and compatible with the cross-linked PLCL matrix, avoiding performance deterioration caused by agglomeration; The cross-linked network was fully formed and structurally stable, providing reliable mechanical support; The dense and defect-free microstructure contributes to the high radial support force, excellent compression recovery, and slow controlled degradation of the stent. In summary, the homogeneous and compact microstructure verified by SEM provides a solid structural basis for the superior mechanical properties, radiopacity, and long-term stability of the esophageal stent.

#### 3.4.4 *In vitro* cytotoxicity test

**FIGURE 13:**
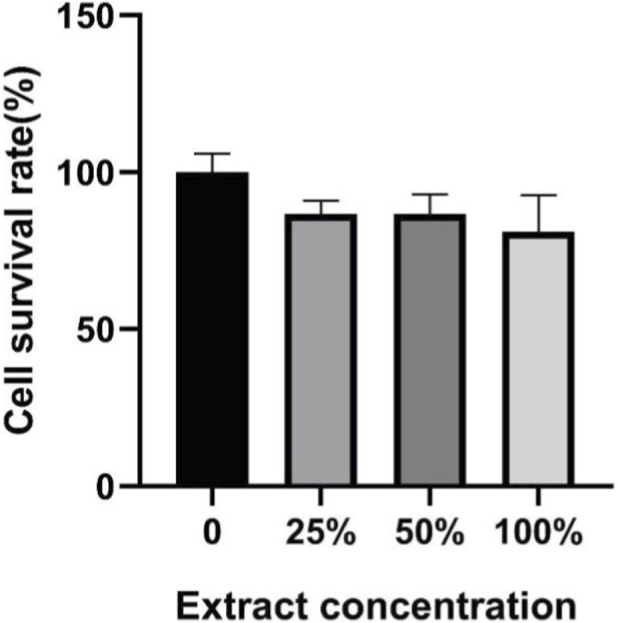
*In vitro* cytotoxicity test.

Biocompatibility is a prerequisite and core requirement for the clinical application of medical stent materials. In this study, cytotoxicity testing was performed in accordance with the ISO 10993–5 (Biological evaluation of medical devices-Part 5: Tests for *in vitro* cytotoxicity) standard. The standard specifies that a material is considered non-cytotoxic if cell viability remains ≥70% after at least 24 h of incubation. The cytotoxicity of the novel polylactic acid-based material was evaluated using the CCK-8 assay. As shown in Figure 13, the viability of L929 mouse fibroblasts cultured for 72 h remained above 70% at three extract concentration gradients (25%, 50%, and 100%), fully meeting the requirements of the ISO 10993–5 standard. These results demonstrate that the novel polylactic acid-based material exhibits no obvious cytotoxicity and possesses favorable biocompatibility, which can effectively avoid complications such as esophageal mucosa injury caused by material cytotoxicity, providing important safety assurance for its clinical application. However, it should be clearly pointed out that this test mainly reflects the biosafety of the material in its initial state, and fails to fully simulate the cumulative exposure effect of continuous degradation products of the material within weeks to months after clinical implantation. In addition, the contrast agent diiodoneopentyl glycol (DINPG) introduced in this study may gradually release iodine-containing fragments during degradation, and its long-term cytocompatibility requires further verification.

#### 3.4.5 Degradation test

**FIGURE 14:**
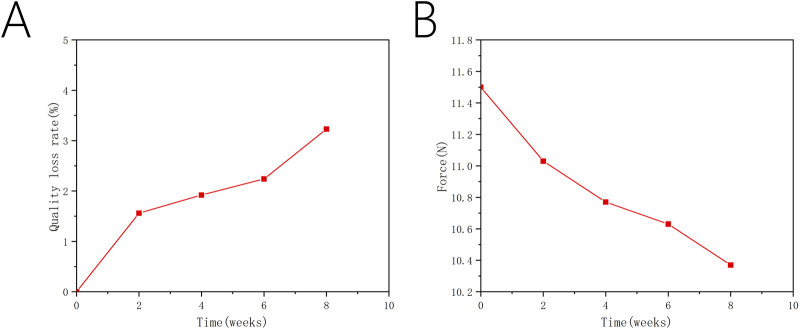
Degradation test **(A)** Mass loss rate; **(B)** Radial support force.

The degradation performance and excellent mechanical stability of biodegradable esophageal stents directly determine their clinical therapeutic efficacy and long-term safety. In this study, an *in vitro* degradation experiment (PBS, 37 °C, pH 7.4) was conducted to monitor the changes in mass and mechanical properties of the stent during an 8-week degradation period, with the results presented in Figure 14. As shown in Figure 14A, the mass loss rates of the stent at 2, 4, 6, and 8 weeks were 1.56%, 1.92%, 2.24%, and 3.23%, respectively, showing a slow and steady degradation trend. The cumulative mass loss rate at 8 weeks was only 3.23%, indicating that the material possesses outstanding mechanical stability and controllable degradation characteristics. As shown in Figure 14B, the average radial support forces of the stent at 0, 2, 4, 6, and 8 weeks of degradation were 11.50 N, 11.03 N, 10.77 N, 10.63 N, and 10.37 N, respectively, displaying an overall slow decreasing trend. More importantly, even after 8 weeks of degradation, the stent still maintained a radial support force above 10 N, retaining 90.17% of its initial support force. This indicates that the stent can still provide sufficient radial support for the stenotic esophageal segment during the 8-week degradation period, without a significant reduction in supporting performance caused by degradation. Furthermore, *in vitro* degradation experiments in this study demonstrated that the stent retained over 90% of its radial support force after 8 weeks, with a mass loss rate of merely 3.23%. According to ESGE guidelines, stents need to maintain effective support for at least 6–8 weeks for refractory benign esophageal strictures, while the commercial biodegradable stent SX-ELLA BD has a support duration of 6–8 weeks. The stent developed in this study exhibited a markedly slower decay rate of support force compared with SX-ELLA BD, and its estimated effective support period could reach 12–16 weeks. This offers a more sufficient time window for tissue remodeling, especially for refractory strictures with severe fibrosis. Nevertheless, a prolonged degradation cycle may raise the risks of stent embedding or tissue hyperplasia, and the matching performance between its *in vivo* degradation behavior and tissue response should be validated in subsequent animal experiments. In summary, the *in vitro* degradation results confirm that the esophageal stent fabricated from the novel polylactic acid-based material exhibits both favorable degradation performance and excellent mechanical stability. It can achieve slow and controllable degradation while continuously providing effective support during the degradation process, meeting the practical requirements for the clinical treatment of esophageal stenosis.

## 4 Conclusion

This study successfully developed a radiopaque biodegradable esophageal stent based on novel polylactic acid materials. Key innovations:

1. A bifunctional iodine-containing monomer (diiodoneopentyl glycol, DINPG) was designed and synthesized, which acts not only as an X-ray contrast agent but also as a cross-linking co-monomer and nucleating agent, synchronously enhancing radiopacity, radial support force, and structural stability of the stent. 2. A cross-linked poly(lactide-co-caprolactone) network with low toxicity catalyst (zinc neodecanoate) was constructed, achieving balanced mechanical strength, elasticity, and biosafety superior to conventional PLA/PCL materials. 3. A bell-shaped flared end plus external spiral texture stent structure was developed, which significantly improves anti-migration performance while maintaining excellent compression recovery (recovery rate >98%). 4. The prepared stent achieves slow controlled degradation (only 3.23% mass loss in 8 weeks) and retains >90% radial support force after 8-week *in vitro* degradation, meeting clinical requirements for long-term effective support.

To verify the reliability of the material synthesis route, multi-scale characterization methods, including FTIR, nuclear magnetic resonance spectroscopy, gel permeation chromatography, scanning electron microscopy, and Micro-CT, were employed to systematically analyze the chemical structure, molecular weight distribution, micromorphology, and radiopacity of the intermediates and final products. The characterization results confirmed the successful preparation of the novel polylactic acid-based material. In addition, static water contact angle measurements and tensile tests demonstrated that the novel polylactic acid-based material possessed a suitable hydrophobic surface (water contact angle >90°) and excellent elastic deformability (elongation at break >600%), meeting the dual requirements of surface properties and mechanical adaptability for esophageal stent applications.

To prepare esophageal stents with both high radial support force and anti-migration performance, the optimal material formulation was determined in this study by adjusting the amount of contrast agent and crosslinking agent. According to radial support force tests, the best formulation was identified as 30% contrast agent loading and 1.25-fold crosslinking agent addition. Based on the conventional straight tubular stent structure, flared ends and an external spiral texture at the mid-section were further designed. Anti-migration force tests verified that the modified stent exhibited favorable anti-migration performance. Meanwhile, compression-release tests and *in vitro* degradation experiments were carried out, which demonstrated excellent mechanical stability and degradation properties of the stent. In addition, cytotoxicity was evaluated using the CCK-8 assay, and the results showed that cell viability was above 70%, indicating good biocompatibility. In summary, this study establishes an integrated strategy for developing radiopaque, biodegradable esophageal stents from material design, synthesis, structure optimization to molding fabrication. The as-prepared stents possess outstanding mechanical properties, favorable biocompatibility, excellent radiopacity, and controllable degradability. Personalized customization of stents can be readily achieved by modifying mold design, showing promising application prospects in the treatment of esophageal stenosis.

This study still has certain limitations: for example, the material strength and long-term stability need to be further improved and verified. In addition, we will continue our research in the future to further investigate the biocompatibility and safety of the material, conduct animal experiments of the esophageal stent, and further improve the performance verification and demonstrate its practical value.

## Data Availability

The original contributions presented in the study are included in the article/supplementary material, further inquiries can be directed to the corresponding authors.

## References

[B1] Arima Y. Iwata H. (2007). Effect of wettability and surface functional groups on protein adsorption and cell adhesion using well-defined mixed self-assembled monolayers. Biomaterials 28 (20), 3074–3082. 10.1016/j.biomaterials.2007.03.013 17428532

[B2] Arima Y. Iwata H. (2015). Preferential adsorption of cell adhesive proteins from complex media on self-assembled monolayers and its effect on subsequent cell adhesion. Acta Biomater. 26, 72–81. 10.1016/j.actbio.2015.08.033 26306676

[B3] Brinkmann F. Uhlig K. Sambale A. Stommel M. Berning M. Babatz J. (2024). Anchoring fins of fully covered self-expandable metal stents affect pull-out force and stent migration. Gastrointest. Endosc. 99 (3), 377–863. 10.1016/j.gie.2023.10.036 37863243

[B4] Ferrari M. Cirisano F. Morán M. C. (2019). Mammalian cell behavior on hydrophobic substrates: influence of surface properties. Colloids Interfaces 3 (2), 48. 10.3390/colloids3020048

[B5] Fujimoto K. L. Yamawaki-Ogata A. Uto K. Usui A. Narita Y. Ebara M. (2021). Long term efficacy and fate of a right ventricular outflow tract replacement using an elastomeric cardiac patch consisting of caprolactone and D,L-Lactide copolymers. Acta Biomater. 123, 222–229. 10.1016/j.actbio.2021.01.022 33476828

[B6] Garbey M. Salmon R. Fikfak V. Clerc C. O. (2016). Esophageal stent migration: testing few hypothesis with a simplified mathematical model. Comput. Biol. Med. 79, 259–265. 10.1016/j.compbiomed.2016.10.024 27825039

[B7] Hanbo H. (2018). Experimental Research on Novel Biodegradable Esophageal Stents. [Doctoral dissertation]. Shanghai Jiao Tong University.

[B8] Hirdes M. M. Vleggaar F. P. De B. M. Siersema P. D. (2013). *In vitro* evaluation of the radial and axial force of self-expanding esophageal stents. Endoscopy 45 (12), 997–1005. 10.1055/s-0033-1344985 24288220

[B9] Huang X. Lu J. An Y. Xu M. Chen X. Liu C. (2025). Electrospun PLGA/PCL nanofiber film loaded with LPA promotes full-layer wound healing by regulating the keratinocyte pyroptosis. ACS Appl. Mater Interfaces 17 (14), 20756–20767. 10.1021/acsami.4c22495 40152284

[B10] Jun E. J. Song H. Y. Park J. H. Bae Y. S. Paulson B. Lee S. (2018). *In vivo* fluorescence microendoscopic monitoring of stent-induced fibroblast cell proliferation in an esophageal mouse model. J. Vasc. Interv. Radiol. 29 (12), 1756–1763. 10.1016/j.jvir.2018.06.024 30266211

[B11] Kim Y. Kwak J. Lim M. Lim S. Y. Chae S. Ha S. J. (2025). Advances in PCL, PLA, and PLGA-based technologies for anticancer drug delivery. Pharmaceutics 17 (10), 1354. 10.3390/pharmaceutics17101354 41155989 PMC12566892

[B12] Li L. Zhang X. Shi J. Chen Y. Wan H. Herth F. J. (2023). Airway stents from now to the future: a narrative review. Respiration 102 (6), 439–448. 10.1159/000530421 37232032

[B13] Li Y. Yuan K. Deng C. Tang H. Wang J. Dai X. (2024). Biliary stents for active materials and surface modification: recent advances and future perspectives. Bioact. Mater 42, 587–612. 10.1016/j.bioactmat.2024.08.031 39314863 PMC11417150

[B14] Li Y. Qiu Y. Wei L. Song Y. Guo W. Yu L. (2025). Enhancing the compatibility and performance of poly (lactic acid) and thermoplastic polyolefin elastomer blends through a dual compatibilization strategy. Int. J. Biol. Macromol. 303, 140513. 10.1016/j.ijbiomac.2025.140513 39892536

[B15] Lin M. Firoozi N. Tsai C. T. Wallace M. B. Kang Y. (2019). 3D-printed flexible polymer stents for potential applications in inoperable esophageal malignancies. Acta Biomater. 83, 119–129. 10.1016/j.actbio.2018.10.035 30366130

[B16] Liu L. L. Qin J. Zeng C. H. Du R. J. Pan T. Ji J. J. (2022). Biodegradable PTX-PLGA-coated magnesium stent for benign esophageal stricture: an experimental study. Acta Biomater. 146, 495–505. 10.1016/j.actbio.2022.04.038 35487426

[B17] Long Z. Wang W. Zhou Y. Yu L. Shen L. Dong Y. (2023). Effect of polybutylene adipate terephthalate on the properties of starch/polybutylene adipate terephthalate shape memory composites. Int. J. Biol. Macromol. 240, 124452. 10.1016/j.ijbiomac.2023.124452 37068541

[B18] Okada G. Matsumoto Y. Habu D. Matsuda Y. Lee S. Osugi H. (2021). Relationship between GLIM criteria and disease-specific symptoms and its impact on 5-year survival of esophageal cancer patients. Clin. Nutr. 40 (9), 5072–5078. 10.1016/j.clnu.2021.08.008 34455266

[B19] Ozdol C. Turhan S. Tulunay C. Altin A. T. Atmaca Y. Candemir B. (2007). Association between proliferative scars and in-stent restenosis. J. Cutan. Med. Surg. 11 (6), 206–210. 10.2310/7750.2007.00039 18042333

[B20] Pan C. Gao Q. Chen Y. Wang Y. Tang Z. (2025). Recent progress in biosourced polylactic acid-based biocomposites for dentistry: a review. Int. J. Biol. Macromol. 310 (4), 143528. 10.1016/j.ijbiomac.2025.143528 40288709

[B21] Reshma C. S. Remya S. Bindu J. (2024). A review of exploring the synthesis, properties, and diverse applications of poly lactic acid with a focus on food packaging application. Int. J. Biol. Macromol. 283 (4), 137905. 10.1016/j.ijbiomac.2024.137905 39577526

[B22] Spaander M. C. W. Van Der Bogt R. D. Baron T. H. Albers D. Blero D. de Ceglie A. (2021). Esophageal stenting for benign and malignant disease: European society of gastrointestinal endoscopy (ESGE) guideline - update 2021. Endoscopy 53 (7), 751–762. 10.1055/a-1475-0063 33930932

[B23] Sun X. Deng X. Yin S. Tang S. Lv L. Tang W. (2025). Mechano-electrochemical synergy of lignin cross-linked PVA gel polymer electrolytes for wide-temperature flexible supercapacitors. ACS Appl. Mater Interfaces 17 (37), 52594–52606. 10.1021/acsami.5c10283 40905745

[B24] Tabares Ocampo J. Marín Valencia V. Robledo S. M. Upegui Zapata Y. A. Restrepo Múnera L. M. Echeverría F. (2023). Biological response of degradation products of PEO-Modified magnesium on vascular tissue cells, hemocompatibility and its influence on the inflammatory response. Biomater. Adv. 154, 213645. 10.1016/j.bioadv.2023.213645 37806213

[B25] Tanaka T. Takahashi M. Nitta N. Furukawa A. Andoh A. Saito Y. (2006). Newly developed biodegradable stents for benign gastrointestinal tract stenoses: a preliminary clinical trial. Digestion 74 (3-4), 199–205. 10.1159/000100504 17341853

[B26] Tiwari S. Liu S. Anees M. Mehrotra N. Thakur A. Tawa G. J. (2023). Quatramer™ encapsulation of dual-targeted PI3-Kδ/HDAC6 inhibitor, HSB-510, suppresses growth of breast cancer. Bioeng. Transl. Med. 8 (5), e10541. 10.1002/btm2.10541 37693068 PMC10487321

[B27] Wang W. Luan Z. Shu Z. Xu K. Wang T. Liu S. (2023). Biosynthetic plastics as tunable elastic and visible stent with shape-memory to treat biliary stricture. Adv. Sci. (Weinh) 10 (29), e2303779. 10.1002/advs.202303779 37552006 PMC10582434

[B28] Yan L. Wang Y. Wang W. Luo J. Cheng B. Yang J. (2024). A poly (lactic-co-glycolic acid) self-pumping janus dressing with bidirectional biofluid transport for diabetic wound healing *via* anti-bacteria and pro-angiogenesis. Int. J. Biol. Macromol. 275 (1), 133361. 10.1016/j.ijbiomac.2024.133361 38960245

[B29] Yang F. Hu Y. Shi Z. Liu M. Hu K. Ye G. (2024). The occurrence and development mechanisms of esophageal stricture: state of the art review. J. Transl. Med. 22 (1), 123. 10.1186/s12967-024-04932-2 38297325 PMC10832115

[B30] Ye G. Zhang X. Bi H. (2024). Construction of high-performance and sustainable polylactic acid composites for 3D printing applications with plasticizer. Int. J. Biol. Macromol. 269 (2), 132162. 10.1016/j.ijbiomac.2024.132162 38723825

[B31] Zhang Y. Xiao J. Fang T. Wei L. Cui W. Zhu Y. (2025a). Degradable biomimetic stent with hydrogel and directional TGF-Loaded nano-fiber membrane for the treatment of esophageal fistula. Biomaterials 328, 123836. 10.1016/j.biomaterials.2025.123836 41197201

[B32] Zhang S. Sun X. Yang X. Fan Y. Liang Y. Li J. (2025b). Research progress on composite nerve guidance conduits with immune-regulatory functions. Front. Immunol. 16, 1622508. 10.3389/fimmu.2025.1622508 40557153 PMC12185515

